# Ultrasound Pretreatment for Enhancing Fine and Ultrafine Flake Graphite Flotation Beneficiation

**DOI:** 10.1021/acsomega.3c09316

**Published:** 2024-02-23

**Authors:** Zheng Tong, Jing Lu, Xinnan Hu, Xiangning Bu, Yujin Sun, Yuran Chen, Saeed Chehreh Chelgani

**Affiliations:** † Key Laboratory of Coal Processing and Efficient Utilization (Ministry of Education, School of Chemical Engineering and Technology, China University of Mining and Technology, Xuzhou 221116, China; ‡ Shandong Polytechnic College,Jining, Shandong 272067, China; § College of Mining Engineering, Taiyuan University of Technology, Taiyuan, Shanxi 030024, China; ∥ State Key Laboratory of Mineral Processing, BGRIMM Technology Group, Beijing 100160, China; ⊥ School of Materials Science and Engineering, Zhengzhou University, Zhengzhou 450001, China; # Minerals and Metallurgical Engineering, Swedish School of Mines, Department of Civil, Environmental and Natural Resources Engineering, Luleå University of Technology, Luleå SE-971 87, Sweden

## Abstract

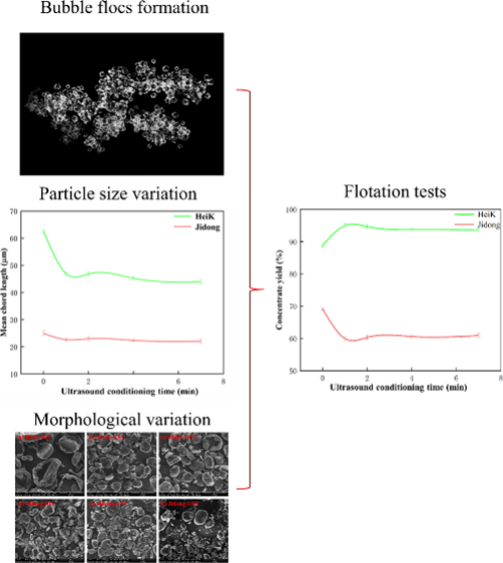

With the severe depletion of coarse flake graphite (a critical raw material) resources, developing and utilizing fine and ultrafine graphite resources have recently attracted attention. Froth flotation is a widely used technique for the initial enrichment of graphite; however, the flotation selectivity decreases significantly along with particle size reduction. Ultrasound pretreatment would be a promising method to improve the flotation of fine particles. As an innovative approach to understand better the flotation response of different flake graphite sizes, this study conducted a comparative analysis based on flotation concentrate yield and ash as well as ash removal rate between the flake graphite with various particle sizes after ultrasound pretreatment. Particle size, X-ray powder diffraction, and scanning electron microscopy and energy dispersive X-ray spectroscopy analyses were used to investigate the effect of ultrasound treatment on mineralogical properties of the flake graphite with varied particle sizes. Process outcomes indicated that the flotation performance of fine flake graphite (mean chord length: 62.63 μm) was significantly enhanced after ultrasound pretreatment. However, flotation of the ultrafine flake graphite (mean chord length: 24.97 μm) after ultrasound treatment was limited due to the difficulty of generating sufficient fragmentation and dissociation by microjets and shock waves formed by the cavitation effect. Compared with conventional flotation, the concentrate yield of ultrasound flotation increased from 88.95 to 94.98%, ash content decreased from 5.72 to 4.87%, and ash removal rate enhanced from 36.94 to 42.61%. Particle size and mineral property analyses confirmed that further crushing and dissociation of the larger flake graphite after ultrasound pretreatment would be the main factors contributing to improved flotation performance. Additionally, the formation of air flocs in the coarse flake graphite during the ultrasound pretreatment process facilitated the flotation recovery of the crushed graphite particles.

## 1 Introduction

Natural graphite, as one of the critical raw materials, can be classified into microcrystalline/aphanitic (amorphous) and crystalline (flake) based on crystallinity as well as grain (flake) size and shape.^1,2^ The grade of flake graphite is generally lower, and its washability is higher than other types,^3^ such as dense crystalline graphite and microcrystalline graphite. By increasing the demand for flake graphite, the beneficiation of fine and ultrafine graphite ores (fine flake and aphanitic graphite) has been widely considered.^3−10^ Flotation and chemical purification (leaching) are the main processes for upgrading graphite and its ash removal.^11,12^ Prior to chemical purification, flotation separation is commonly used for the preliminary enrichment of raw graphite ore with the highest amount of graphite, ∼95%.^11^

However, with the decrease in particle size, graphite flotation efficiency deteriorated due to the low collision probability between particles and bubbles.^13,14^ High entrainment is another remarkable challenge for fine graphite flotation.^15,16^ The development of different flotation reagents (collectors, frothers, depressants, activators, and pH regulators) has been the major focus of investigations to enhance graphite flotation enrichment.^17^ It was reported the ultrasound-assisted technique could be a promising method to enhance the graphite flotation performance. Ultrasound-assisted flotation can be attributed to factors such as the removal of slime coatings and oxidation films attributed to the graphite surface, scrubbing ultrafine impurities, desulfurization, generation of tiny bubbles, dispersion of flotation reagents, aggregation, and breaking locked particles.^18−20^ The breakup of particles by the ultrasound-assisted technique would enhance the separation process, such as flotation,^18−20^ leaching responses,^30^ and preparation of graphene.^122^

Letmathe et al.^21^ reported that using ultrasound during flotation can lead to emulsification of the reagents, dispersion of the solids in the liquid, and improvement of the process efficiency. Kang and Li^22^ found that intensifying graphite flotation by ultrasound treatment can shorten the graphite cleaning process. In addition, acoustic cavitation is one of the standard methods to generate nanobubbles (Bu and Alheshibri, 2021). However, Li et al. (2020) considered that ultrasonication has a negligible influence on the interaction between graphite particles and flotation performance. The contradictory results in ultrasound-assisted graphite can be attributed to the differences in the properties of graphite ores (carbon content and particle size), flotation reagents, and ultrasound parameters (Table 1). It is important to note that there is a concept of “critical grain size”; below that size, the ultrasound-assisted technique is not effective in improving leaching efficiency (45 μm for CuO) (Swamy and Narayana, 2001). Thus, particle size is expected to be essential in ultrasound graphite flotation performance.

**Table 1:** Summary of the Properties of Graphite Ores, Flotation Reagents, and Ultrasound Devices in Ultrasound-Assisted Graphite Flotation

graphite ore					ultrasound device				
source	carbon content (%)	*d*_50_ (μm)	yield (%)	recovery (%)	Brand, model, and type (horn or bath)	frequency (kHz)	power (W)	pretreatment time (min)	ref
Luobei, China^a^	90.56	73	91.46	96.12	not specified, JAC-5500, not specified	25	0–2000	0–6	(22)
NGS Trading & Consulting Corporation, Germany^b^		25		38	Bandelin, RK100H, bath	35		2	(23)
Toamasina Province, Madagascar^c^	10.79	150	10.5	82.42	LABMAN, LMUC-16, bath	40	300	5	(24)

a Kerosene (collector); secondary octyl alcohol (frother).

b Diesel oil (collector); methyl isobutyl carbinol (frother).

c No use (collector); no use (frother).

In general, flake graphite has been classified into coarse (+150–850 μm in diameter), fine (+45–150 μm), and ultrafine (−45 μm) particles (Chelgani et al., 2015). However, few studies have examined the metallurgical response of different-sized graphite ores to ultrasound-assisted flotation. To address this gap, in this study, ultrasound pretreatment was used on fine (mean chord length is 62.63 μm) and ultrafine (mean chord length is 24.97 μm) flake graphite ores and compared their respective flotation performances with and without ultrasound pretreatment. Particle size measurements, ash content, XRD, SEM-EDS, and other relevant parameters were considered to compare and analyze the flotation responses of different size fractions. The outcomes of this investigation would shed new insights into the potential application of ultrasound-assisted flotation for enhancing graphite ore beneficiation.

## 2 Materials and Methods

### 2.1 Flake Graphite

The materials used in this experiment were two different sizes of preliminary flotation graphite concentrates from Heilongjiang Province. The study included two samples, Jidong and HeiK, and the properties of the samples are shown in Table 2. Jidong has an original ore ash content of 16.23%, and HeiK has an original ore ash content of 8.06%. With respect to particle size, the mean chord length of HeiK was 62.63 μm, while that of Jidong was only 24.97 μm. These materials were used as flotation feed in the experiment and will be analyzed in subsequent research.

**Table 2:** Ash Content and Particle Size of the Graphite Samples

graphite samples	ash content, %	mean chord length, μm
HeiK	8.06	62.63
Jidong	16.23	24.97

### 2.2 Flotation Tests

An ultrasound crusher (VCX800; Sonics, American, 800 W) and an RK/FD type 0.5 L flotation cell (with a power of 120 W and an impeller diameter of 45 mm) were employed. The ultrasound crusher was used for graphite pretreatment, while the flotation cell was used in the flotation experiments.

**Figure 1:**
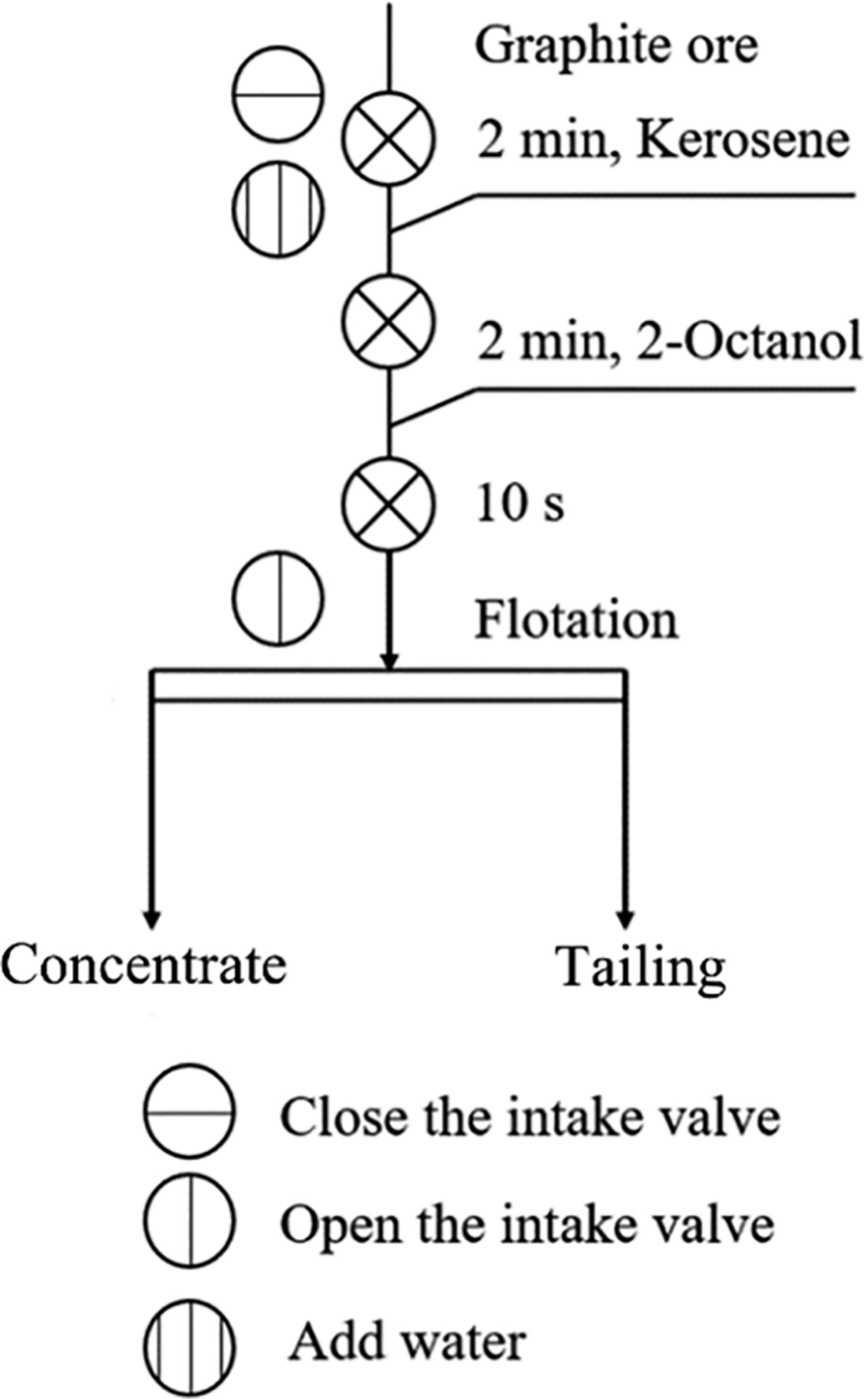
Diagram of graphite flotation.

This ultrasound crusher has a probe attached with a tip of 13 mm, length of 136 mm, and weight of 340g and made of titanium alloy. For ultrasonic pretreatment, the probe was submerged vertically below 50 mm of the pulp surface, and then, the ultrasound crusher started to work. Graphite pulp (pulp volume 300 mL, concentration 100 g/L) with different particle sizes was pretreated with ultrasound for 0, 1, 2, 4, and 7 min. Subsequently, flotation experiments were conducted, and the concentrate yields corresponding to the flotation times were recorded. Various ultrasound powers (0, 20, 40, 60, 80, and 100 W) were used to assess their effects on ash removal through a constant treatment time. Sinopharm Group Co. Ltd., China, supplied the collector (kerosene) and frother (secondary octanol) used in the experiments. All tests were carried out using tap water, with a natural pH level. The collector and frother dosages were 100 and 70 g/t for larger flake graphite (HeiK) flotation and 200 and 140 g/t for smaller flake graphite (Jidong) flotation, respectively. The pulp concentration was kept at 60 g/L, the flotation cell speed was set to 2000 r/min, and the air charge was 250 L/h. The specific steps for conducting the flotation can be seen in Figure 1. Ash content analyses were conducted on two graphite raw ores (RO), conventional flotation concentrate (CFC), ultrasound flotation concentrate (UFC), and flotation tailings under the highest yield conditions. The ash removal rate α can be calculated as follows:
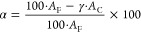
1where *A*_F_ is the ash content of the raw ore, %, γ is the concentrate yield, %, and *A*_C_ is the ash content of the concentrate, %.

### 2.3 XRD and SEM-EDS measurements

The mineralogical characterizations of the graphite samples were assessed using XRD (6100, Shimadzu Corporation, Japan). The XRD experiment was carried out at a 40 kV accelerating voltage and 30 mA current while utilizing a Cu Kα radiation source. The scanning speed and scan range were set at 8°/min and 10–90°, respectively. SEM-EDS (FIB-SEM, Helios G4 CX, Thermo Fisher Scientific) was used to characterize the morphology and impurity distribution of the graphite samples. The acceleration voltage was 10 kV, and the operating current was 5.5 nA. The SEM-EDS images were calibrated to enhance the results’ clarity.^9,25^

### 2.4 Particle Size Measurement

A Focused Beam Reflectance Measurement “FBRM” (G400, Mettler Toledo, USA) system was used to measure the particle size. The working principle underlying FBRM can be found in the literature.^26^ A graphite suspension was prepared by the same method used for the flotation test, placed on the FBRM–PVM system, and stirred using a magnetic stirrer at 400 r/min while the FBRM–PVM system operated.^8,27−29^

## 3 Results and Discussion

### 3.1 Effect of Ultrasound Pretreatment on the Flotation Yield

#### 3.1.1 Ultrasound Time

**Figure 2:**
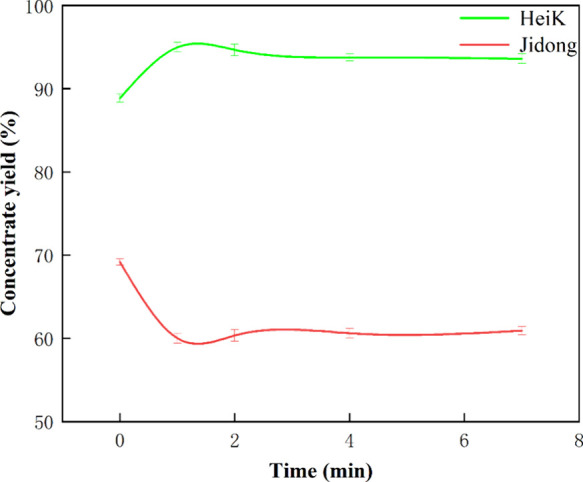
Graphite concentrate yield during different ultrasound conditioning times (constant ultrasound power: 100%).

Experimental outcomes (Figure 2) indicated that after ultrasound pretreatment, the concentrate yield of HeiK increased to varying degrees with different pretreatment times. The concentrate yield showed a trend of increasing and then decreasing with time, and the highest concentrate yield was observed at 1 min. The increase in concentrate yield highlighted that ultrasound pretreatment could promote the flotation of fine flake graphite. In contrast, the concentrate yield of Jidong decreased after ultrasound pretreatment, and the concentrate yield basically showed a gradual decrease with time. This decrease in concentrate yield demonstrated that ultrasound pretreatment suppressed the flotation of ultrafine flake graphite. In other words, these results suggested that ultrasound pretreatment can promote fine flake graphite flotation while inhibiting ultrafine flake graphite’s flotation. The difference in flotation yield between the two graphite samples increased from 19% (without ultrasound pretreatment) to the maximum value of 35% (1 min ultrasound pretreatment).

#### 3.1.2 Ultrasound Power

**Figure 3:**
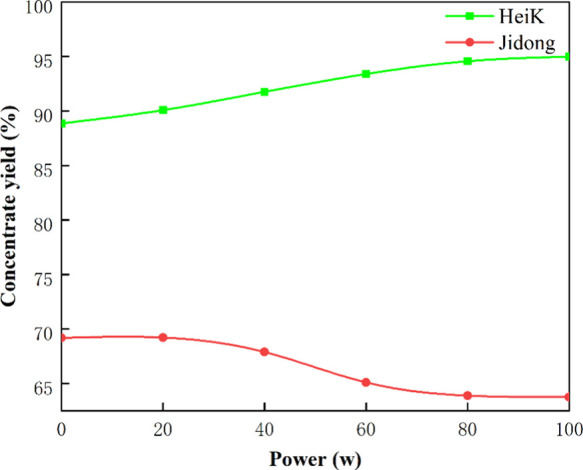
Graphite concentrate yield through different ultrasound power treatments (1 min).

Exploring the effect of various ultrasound powers during 1 min treatment demonstrated (Figure 3) that HeiK’s concentrate yield increased at different ultrasound powers. The concentrate yields showed a gradual increase with enhancing ultrasound power. In contrast, Jidong’s concentrate yield decreased after ultrasound pretreatment and gradually decreased with increasing ultrasound power. It was found that ultrasound pretreatment had varying effects on the two graphite samples with different particle sizes. Furthermore, the difference between the flotation concentrate yields of the two graphite samples increased with increasing ultrasound power and reached its maximum at 100 W. This indicates that the effectiveness of ultrasound treatment varies for graphite samples of different particle sizes. Ultrasound treatment can enhance the concentrate yield of certain graphite samples, but it may lead to a decrease in concentrate yield for other graphite. Therefore, when applying ultrasound treatment in practical applications, it is important to consider the characteristics of the graphite samples and the suitability of ultrasound treatment.

### 3.2 Effect of Ultrasound Pretreatment on the Particle Size Distribution of Graphite

#### 3.2.1 Ultrasound time

**Figure 4:**
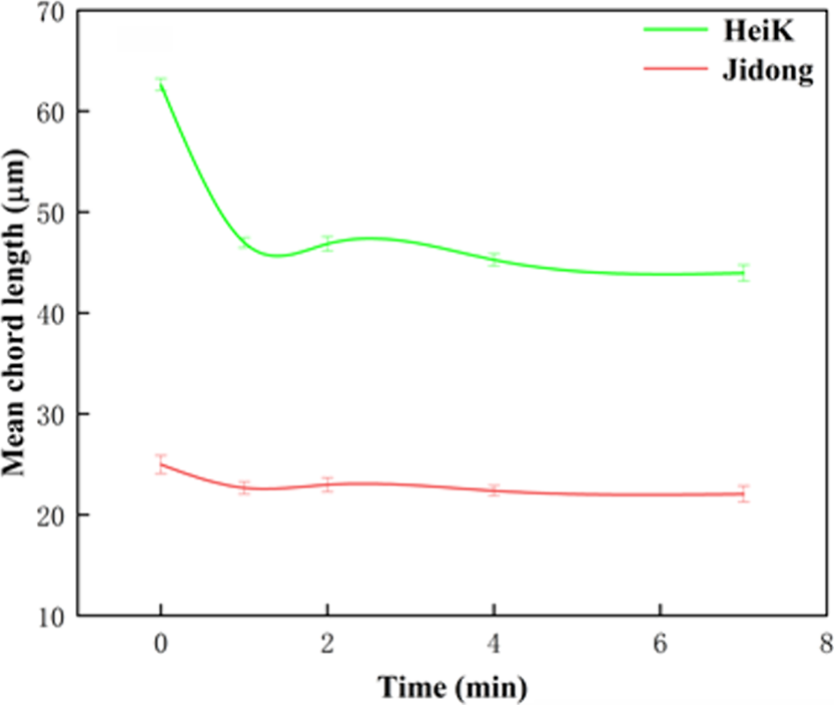
Graphite particle size variation at different ultrasound times.

Exploring the variation of graphite particle sizes at a constant ultrasound power of 100% and different ultrasound times revealed that the particle sizes of both graphite types were reduced after the ultrasound pretreatment (Figure 4). Additionally, the particle sizes of larger flake graphite tended to decrease, and the reduction in particle size was more distinct, with the mean chord length decreasing from 62.63 to less than 50 μm after ultrasound pretreatment. In contrast, the particle size of ultrafine flake graphite decreased to a lesser extent. These observations indicated that the bubble cavitation caused by ultrasound pretreatment leads to a decrease in graphite particle sizes, and the effect is more pronounced for larger flake graphite. The reduction in the particle size of larger flake graphite after ultrasound pretreatment can be attributed to the phenomenon of bubble cavitation. This occurs when rapid pressure changes induced by ultrasound waves cause bubbles to form and collapse within the liquid medium. The resulting localized high-intensity forces can physically disrupt and break down the graphite particles, leading to a reduction in particle size. As a result, larger flake graphite particles tend to experience a more significant reduction in size than ultrafine flake graphite particles. Additionally, the structure of the graphite particles, such as the degree of crystallinity and the presence of impurities, can also affect the response to ultrasound treatment. The Jidong graphite samples contain higher impurities, which have a higher hardness compared to graphite. Therefore, it is more difficult for these impurities to be broken down under the action of ultrasound.

After exceeding 1 min of ultrasound pretreatment, the particle size of the larger flake graphite did not change significantly. This meant that further extending the ultrasound pretreatment time could not further significantly dissociate the graphite particles and would be wasting energy. These results are consistent with the former outcomes (Figure 2), where the best flotation yield for the larger flake graphite was obtained after 1 min of ultrasound pretreatment, and extending the ultrasound pretreatment time could not further improve the flotation concentrate yield.

#### 3.2.2 Ultrasound Power

**Figure 5:**
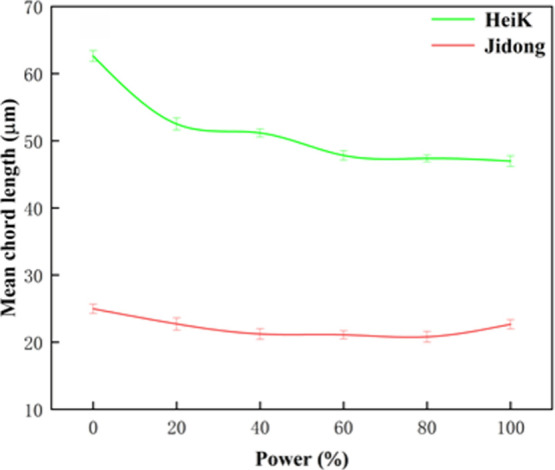
Graphite particle size variation at different ultrasound powers.

Figure 5 displays the particle size variation (characterizing particle size using average chord length) of the two graphite samples at different ultrasound powers (1 min treatment). Results showed that the particle sizes of both graphite samples (HeiK and Jidong) were reduced after ultrasound pretreatment, but the degree of reduction was varied. When the ultrasound power increased from 0 to 60 W, the particle size reduction of HeiK was significant, and the particle size change was minimal when the ultrasound power continued to increase. These results suggested that the energy provided by the ultrasound power of 60 W has reached the breaking energy required for most of the fine flake graphite particles. When the ultrasound power was increased from 0 to 100 W, HeiK particle sizes decreased more substantially, from 62.63 to 46.98 μm (crushing ratio of 1.33). In contrast, Jidong particle sizes decreased less from 24.97 to 22.66 μm (crushing ratio of 1.1). These outcomes signified that during the ultrasound pretreatment process, the jets generated by the collapse of microbubbles caused the graphite particles to be crushed and dissociated, resulting in a decrease in particle size.^18,30,31^ Additionally, ultrasound pretreatment is more effective in reducing the particle size of larger flake graphite.

### 3.3 Conventional Flotation vs Ultrasound Flotation

Ash content analyses illustrated (Table 3) that after ultrasound flotation (ultrasound treatment 1 min, power 100 W), the ash content of HeiK’s concentrate was significantly reduced from 8.06 to 4.87%, which was a remarkable ash reduction effect. The ash content was even lower than the ash content of 5.72% observed in the concentrate obtained from the conventional flotation. Moreover, the concentrate yield of 94.98% obtained by ultrasound flotation was much higher than the 88.85% yield achieved by conventional flotation. The ash content of the tailings resulting from ultrasound flotation also showed impressive improvement, increasing from 21.29% observed in conventional flotation to 43.40%. In addition, the combustible recovery increased from 91.11% in conventional flotation to 98.28% for ultrasound-treated samples. Thus, for HeiK’s concentrate, the ash content decreased by 0.85%, the concentrate yield increased by 6.13%, and the tailing ash content further increased after ultrasound pretreatment, significantly improving the flotation performance.

**Table 3:** Results of Conventional Flotation and Ultrasound Flotation Experiments

samples		concentrate yield/%	ash content of concentrate/%	ash content of tailing/%	ash removal rate/%	combustible recovery/%
RO	Jidong		16.23			
	HeiK		8.06			
conventional flotation	Jidong	69.22	10.98	26.69	53.17	73.56
	HeiK	88.85	5.72	21.29	36.94	91.11
ultrasound flotation	Jidong	69.18	10.22	23.25	56.43	74.14
	HeiK	94.98	4.87	43.40	42.61	98.28

For Jidong, after ultrasound flotation, the ash content of the concentrate decreased from 10.98% in conventional flotation to 10.22%. However, the concentrate yield slightly decreased, from 69.22 to 69.18%. Meanwhile, the tailings’ ash content was reduced from 26.69 to 23.25% after ultrasound flotation. Therefore, the ultrasound pretreatment did not improve the flotation performance of Jidong, and some low-ash concentrates were lost in the tailings.

Regarding the ash removal rate, for Jidong, the ash removal rate after ultrasound flotation was slightly higher than that observed in conventional flotation (53.17% vs 56.43%). Nevertheless, this improvement was achieved at the expense of losing a portion of the concentrate yield. Conversely, for HeiK, the ash removal rate obtained through ultrasound flotation was 42.61%, which was higher than that achieved by conventional flotation (36.94%) by 5.67%. Additionally, the ash content of the concentrate resulting from ultrasound flotation was lower, indicating that the ash removal was more effective.

### 3.4 XRD and SEM-EDS Analyses

**Figure 6:**
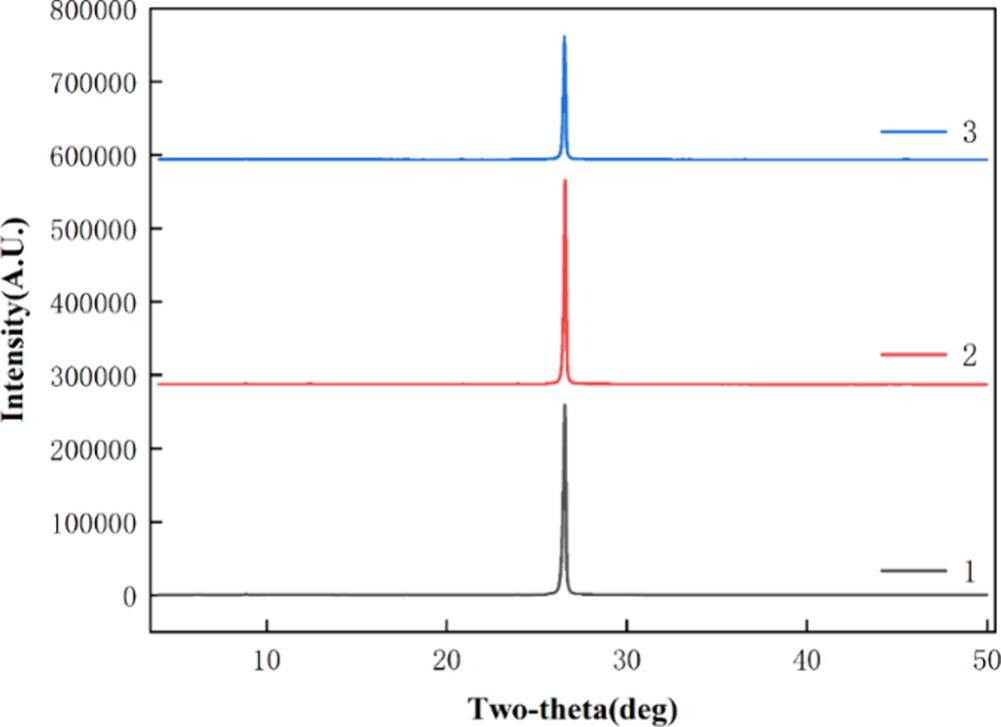
XRD pattern of the HeiK graphite (1-RO; 2-CFC; 3-UFC).

**Figure 7:**
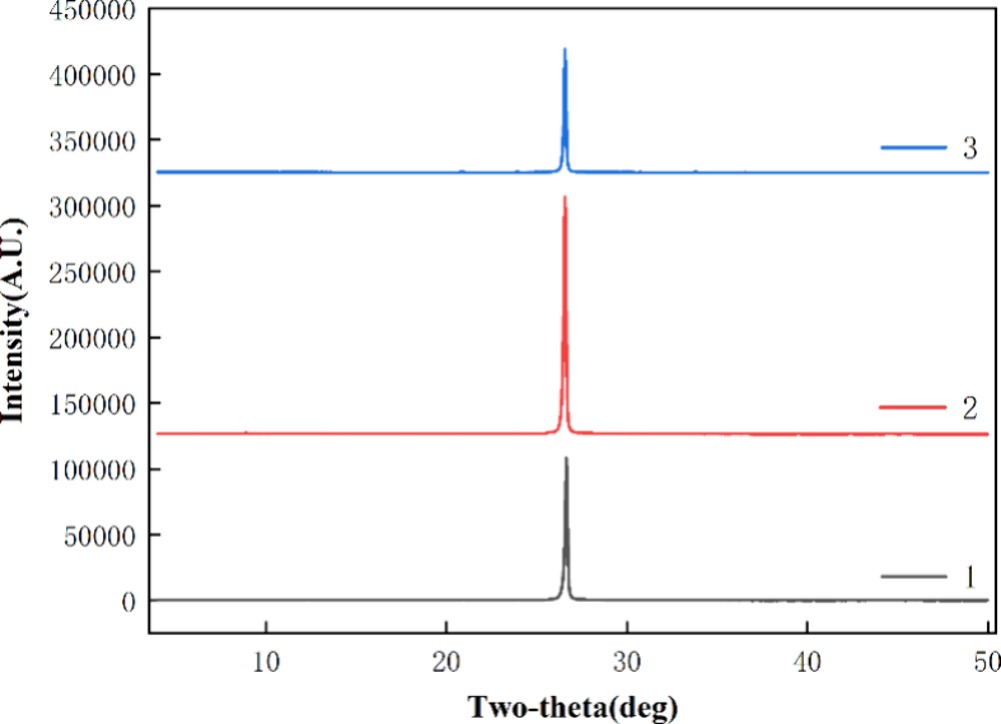
XRD pattern of the Jidong graphite (1-RO; 2-CFC; 3-UFC).

XRD results (Figure 6 and Figure 7) showed that the main mineral component of the raw ores was graphite. The intensity of peaks observed in the XRD analysis is reduced after the ultrasound treatment. This can be attributed to various factors, including the disruption of the crystalline structure due to mechanical stress and shear forces caused by ultrasound, particle size reduction leading to a decrease in the number of crystal domains contributing to the diffraction signal, and the formation of defects or amorphous regions in the graphite structure. After the treatment of conventional flotation and ultrasound flotation, the peak shape and the position of the peaks did not change, which indicated that the crystal structure of graphite had no change. Compared with other minerals, graphite’s more stable crystal structure gives it higher electrical and thermal conductivity, making it a highly valuable material. Therefore, it is essential to maintain the crystal structure of graphite while improving beneficiation efficiency, and both conventional flotation and ultrasound flotation can meet this requirement. In summary, XRD results demonstrated that the stability of the graphite crystal structure was maintained, indicating that both conventional flotation and ultrasound flotation are effective methods for graphite beneficiation.

**Figure 8:**
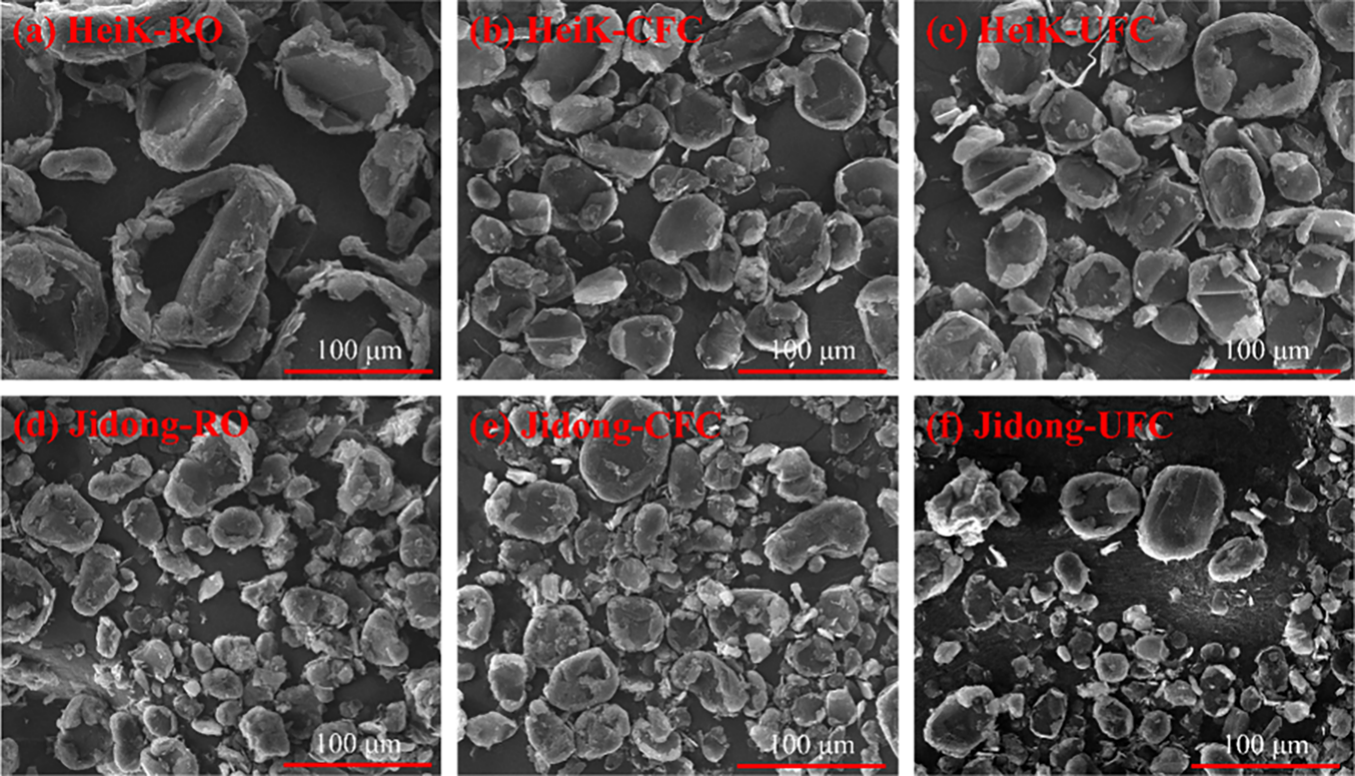
SEM images of (a–c) HeiK and (d–f) Jidong graphite samples of the RO, CFC, and UFC.

As known from the literature, ultrasound generated sparse, high-frequency waves that propagate through the pulp, inducing reciprocating vibration of the liquid at high speeds.^32^ In the negative pressure zone of the vibration, the surrounding liquid could not replenish quickly enough, forming myriad tiny vacuum bubbles. In the positive pressure zone, the tiny bubbles rapidly collapse under pressure, generating a powerful impact wave due to the mutual collision between the liquids.^33,34^ These results in an instantaneous high pressures of up to several thousand atmospheres.^30,35^ The continuous occurrence of instant high pressures produces a series of small “explosions,″ continuously impacting the graphite surface. This process removes the mineral cover and achieves the role of cleaning the surface of graphite particles.^36−38^ The SEM images (Figure 8) of the RO, CFC, and UFC for both graphite ores at the highest yield conditions support this information from the literature.

Additionally, Figure 8 indicates that ultrasound treatment could generate finer particles, which is favorable for the dissociation of graphite and gangue minerals. Figure 8d–f shows that the particle size of the Jidong changed negligibly after ultrasound treatment, which was consistent with the pattern observed in Figure 5. Compared to the Jidong sample, the ultrasound fragmentation effect was more significant for the HeiK sample (Figure 8a–c), which had a larger particle size. The results displayed in the SEM images are consistent with the findings in Figures 4 and 5.

**Figure 9:**
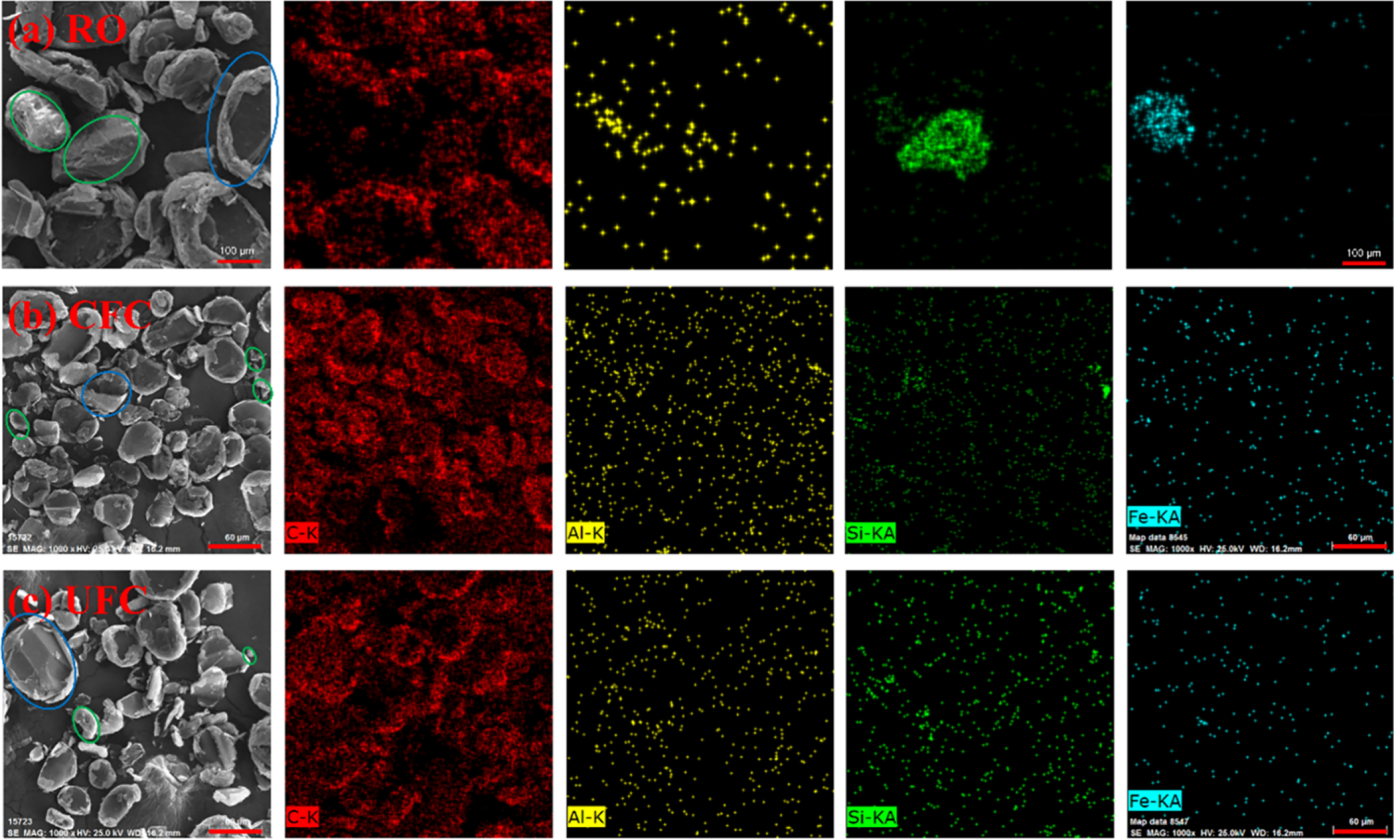
SEM-EDS analysis of the HeiK graphite: (a) RO, (b) CFC, and (c) UFC.

**Figure 10:**
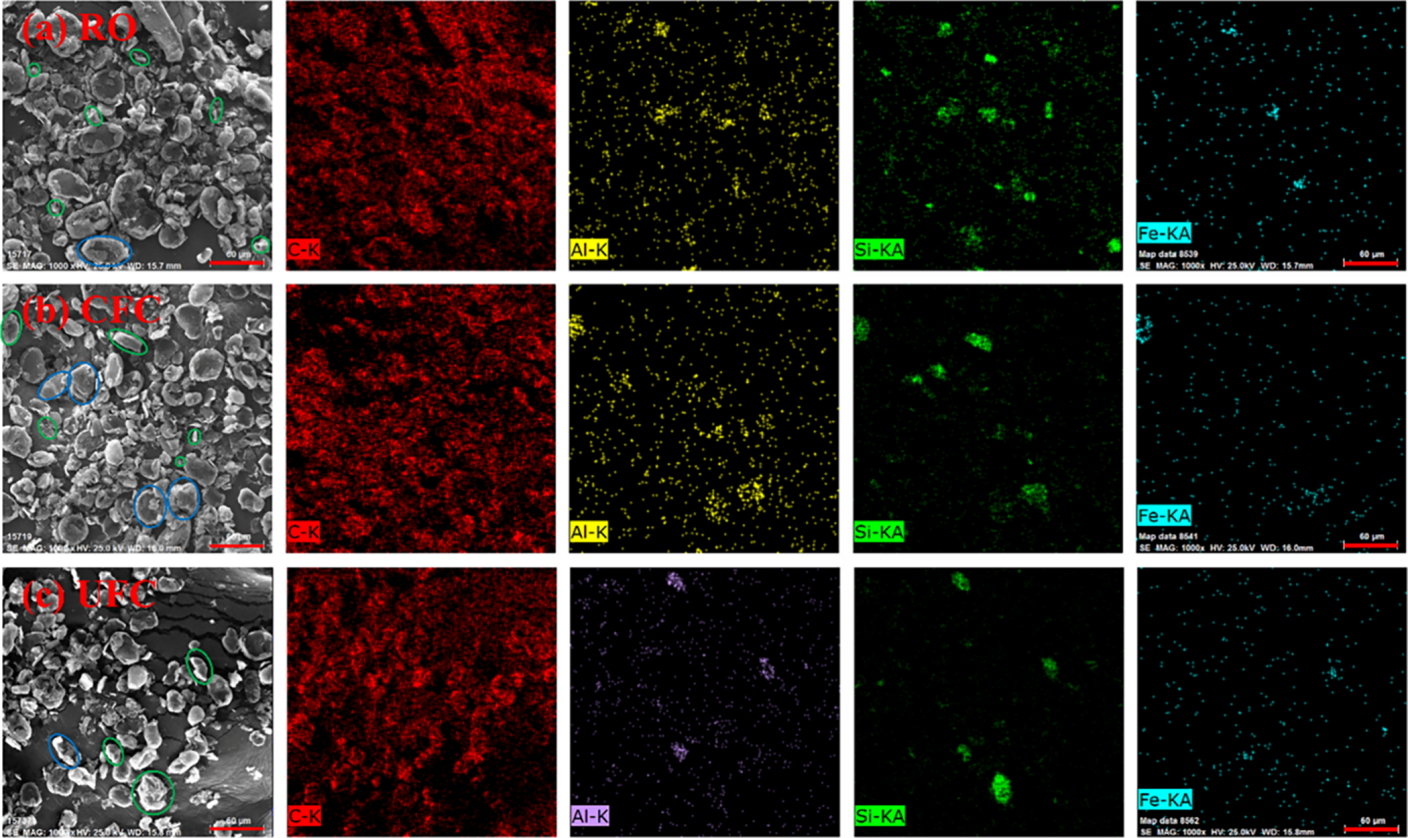
SEM-EDS analysis of the Jidong graphite: (a) RO, (b) CFC, and (c) UFC.

The SEM-EDS images (Figures 9 and 10) of the RO, CFC, and UFC of HeiK and Jidong graphite indicated that for both graphite samples, gangue particles in a monomer dissociation state could be observed in both CFC and UFC. These gangue particles were mixed into the flotation concentrates through mechanical entrainment (indicated by the green circles). Some of the dissociated gangue particles exist in the original ores, while others might have been broken and dissociated by shock waves and microjets generated during the ultrasound cavitation process, or they might have been removed from the surface of graphite by the cleaning effect.^39,40^ These analyses (Figures 9 and 10) highlighted that graphite and gangue phases were in an intergrowth state (indicated by blue circles). In the HeiK sample, gangue particles were relatively concentrated and distributed at the edges of large particles, which is conducive to further dissociation between graphite and gangue minerals through the ultrasonic crushing effect. However, in the Jidong sample, the intergrowth state of gangue minerals in the graphite-gangue mineral intergrowth body was more dispersed, which required reducing the particle size to a sufficiently finer size for effective dissociation between graphite and gangue minerals. Nevertheless, a critical particle size exists for ultrasonic crushing, beyond which the particle size is difficult to reduce further under the ultrasonic effect.^31,41^ This is why the flotation performance of the Jidong sample after ultrasonic treatment did not significantly improve as high as the HeiK sample.

### 3.5 Mechanism Analysis

After ultrasound pretreatment, two graphite ores with different particle sizes displayed varied flotation results. HeiK exhibited a significant reduction in particle size, increased flotation concentrate yield, and decreased ash content after ultrasound pretreatment. In contrast, Jidong showed only a slight reduction in particle size, a minor decrease in the ash content of the flotation concentrate, and a decrease in yield after ultrasound pretreatment. The Jidong’s raw ore was classified as ultrafine particle size, with the mean chord length value further reduced to below 23 μm after ultrasound pretreatment. While crushing and dissociating graphite particles can help separate graphite from gangue minerals and facilitate the removal of ash content in the concentrate, the probability of collision between particles and air bubbles markedly dropped with particle size reduction, impacting the flotation separation. Various investigations have shown that medium-sized particles could lead to the highest flotation performance, while microparticles result in lower selectivity and low collision and adhesion probability, leading to lower flotation efficiency.^42,43^

The reasons for the increase in concentrate yield of larger flake graphite (HeiK samples) during the ultrasound flotation process could be a combination of different scenarios. The first scenario is that ultrasound pretreatment assisted the dissociation of graphite particles from gangue minerals, decreasing particle size. This is evident in particle size analysis and SEM-EDS conducted on graphite samples following ultrasound pretreatment. In the case of larger flake graphite, this reduction in particle size led to an improved flotation performance due to its enhanced selectivity and collision efficiency with bubbles. Such improvements have been reported in other studies on ultrasonic-assisted flotation of potash ores,^44^ spodumene,^37^ and coals.^19,20,45,46^ Moreover, ultrasound pretreatment has been widely reported to have a cleaning effect on mineral particles. When ultrasound is applied, the cavitation bubbles generated can break down mineral particles and remove them from the surface of graphite particles. Exposure to fresh graphite surfaces increases the number of active sites available for interaction with collectors during the flotation process, improving hydrophobicity and better separation efficiency. Additionally, ultrasound can enhance the dispersion of collectors and provide better coverage of the graphite surface, further contributing to improving flotation performance.

Another scenario is that ultrasound pretreatment effectively forms air flocs consisting of aggregates of graphite particles, air bubbles, and agents. These air flocs have been observed during the ultrasound process, and several studies have confirmed their formation.^36,47−50^ Zhang et al. (2022) reported that the application of ultrasound created air flocs that consisted of graphite particles, air bubbles, and reagents, which increased the floatability of graphite particles. The formation of air flocs is critical in the flotation process as it facilitates adhesion between graphite particles and air bubbles, promoting premineralization and direct up-floating of graphite particles into the concentrate. The air flocs act as a carrier for the graphite particles, allowing them to be transported more effectively to the surface of the flotation cell. This is attributed to the ability of the air flocs to provide an additional contact opportunity between graphite particles, air bubbles, and collectors, thereby improving the probability of their interaction.

Ultrasound waves generate mechanical pressure waves that induce cavitation in the pulp, creating microbubbles and generating local turbulence. This creates violent fluctuations in the pulp level, resulting in the suction of air into the pulp. The high-intensity ultrasound waves create a vibrating motion that generates bubbles in the pulp, which adhere to the graphite particles through collision. These bubbles’ formation leads to air flocs, which consist of aggregates of graphite particles, air bubbles, and reagents. The mechanism behind the generation of air flocs during the ultrasound pretreatment process can be explained by several factors. First, the microbubbles generated during the cavitation process provide a larger surface area for the adsorption of reagents such as collectors and frothers, enhancing their ability to attach to the graphite surface. Additionally, the microbubbles improve the hydrophobicity of the graphite surface by reducing the contact angle between the graphite particles and water. This enhances the adhesion of the particles to the air bubbles, promoting the formation of air flocs.

Furthermore, ultrasound waves have been shown to induce physical shock and shear forces in the pulp, which can break down agglomerates and disperse particles, leading to more effective particle–bubble collision during the flotation process. This facilitates the formation of air flocs in the pulp, leading to improved flotation recovery. Overall, the generation of air flocs during the ultrasound pretreatment process results from many factors working together, including the production of microbubbles, improved adsorption of reagents, and enhanced particle–bubble collision. These factors are critical in achieving efficient flotation separation of graphite.

## 4 Conclusions

Through a comparative analysis based on flotation concentrate yield and ash as well as ash removal rate, the effect of ultrasound pretreatment on the flotation of the flake graphite with different particle sizes was investigated. In addition, the mineralogical properties of the flake graphite were also analyzed to better understand the effect of ultrasound treatment. The main conclusions obtained are as follows.

1. The outcomes of ultrasound-assisted flotation for fine (Heik) and ultrafine (Jidong) graphite samples were reversed. As the ultrasound power increased (0–100 W), the flotation concentrate yield of HeiK gradually enhanced, while the results of ultrasound-assisted flotation for Jidong samples showed an opposite trend. Ultrasound pretreatment enhanced the flotation of larger flake graphite (Heik) while inhibiting that of smaller flake graphite (Jidong). The difference in flotation concentrate yield between the two graphite samples reached a maximum of 35% after 1 min of ultrasound pretreatment.
2. Ultrasound pretreatment reduced the particle size for both graphite samples, although the degree of reduction was varied. Larger flake graphite displayed a more pronounced reduction in particle size. As the ultrasound power increased from 0 to 100 W, HeiK’s particle size decreased from 62.63 to 46.98 μm, representing a crushing ratio of 1.33, while Jidong particle sizes decreased less from 24.97 to 22.66 μm.
3. In addition, the increase in ultrasonic pretreatment time led to further reductions in graphite particle size. However, the reduction in particle size was more significant within 1 min. These reductions were due to the jets generated by the collapse of microbubbles during the ultrasonic pretreatment process, leading to the fragmentation and dissociation of the graphite particles.
4. In comparison to conventional flotation, HeiK’s concentrate ash content decreased by 0.85%, concentrate yield increased by 6.13%, and tailing ash further increased after ultrasound pretreatment, indicating a substantial improvement in flotation performance by ultrasound pretreatment. In contrast, although the ash content of the Jidong concentrate was reduced after ultrasound pretreatment, from 10.98 to 10.22%, the concentrate yield was reduced. The flotation performance did not improve, and a portion of the low-ash concentrate was lost in the tailing.
5. The main reason for the deterioration of Jidong’s ultrasound flotation performance was that ultrasound further reduced the graphite particle size, and the microfine particles had low flotation efficiency due to poor selectivity and low probability of collision and adhesion. The reasons for enhancing the ultrasound flotation performance of larger flake graphite included the dissociation of gangue phases from the surface of graphite particles, size reduction, and the formation of stable air flocs.

## References

[ref1] Andersen S. Z.; Čolić V.; Yang S.; Schwalbe J. A.; Nielander A. C.; McEnaney J. M.; Enemark-Rasmussen K.; Baker J. G.; Singh A. R.; Rohr B. A.; Statt M. J.; Blair S. J.; Mezzavilla S.; Kibsgaard J.; Vesborg P. C. K.; Cargnello M.; Bent S. F.; Jaramillo T. F.; Stephens I. E. L.; Nørskov J. K.; Chorkendorff I. A rigorous electrochemical ammonia synthesis protocol with quantitative isotope measurements. Nature 2019, 570, 504–508. 10.1038/s41586-019-1260-x. 31117118

[ref2] Li H.; Feng Q.; Ou L.; Long S.; Cui M.; Weng X. Study on washability of microcrystal graphite using float–sink tests, International Journal of. Mining Science and Technology 2013, 23, 855–861. 10.1016/j.ijmst.2013.10.012.

[ref3] Sun K.; Qiu Y.; Zhang L.; Liu Q.; Mao Z.; Qian Y. Enhanced fine flake graphite flotation and reduced carbon emission by a novel water-in-oil kerosene emulsion. Colloids Surf., A 2022, 650, 129603 10.1016/j.colsurfa.2022.129603.

[ref4] Chongliang T.; Fangyuan M.; Tingyu W.; Di Z.; Ye W.; Mingjiao L.; Xiangwei L.; Xinyue L. Study on surface physical and chemical mechanism of nanobubble enhanced flotation of fine graphite. J. Ind. Eng. Chem. 2023, 122, 389 10.1016/j.jiec.2023.02.039.

[ref5] Zhao S.; Cheng S.; Xing B.; Ma M.; Shi C.; Cheng G.; Meng W.; Zhang C. High efficiency purification of natural flake graphite by flotation combined with alkali-melting acid leaching: application in energy storage. Journal of Materials Research and Technology 2022, 21, 4212–4223. 10.1016/j.jmrt.2022.11.001.

[ref6] Qiu Y.; Mao Z.; Sun K.; Zhang L.; Yang L.; Qian Y.; Lei T. Cost-efficient clean flotation of amorphous graphite using water-in-oil kerosene emulsion as a novel collector. Advanced Powder Technology 2022, 33, 103770 10.1016/j.apt.2022.103770.

[ref7] Bu X.; Zhang T.; Chen Y.; Peng Y.; Xie G.; Wu E. Comparison of mechanical flotation cell and cyclonic microbubble flotation column in terms of separation performance for fine graphite. Physicochem. Probl. Miner. Process. 2018, 54, 732–740. 10.5277/ppmp1873.

[ref8] Gao J.; Bu X.; Zhou S.; Wang X.; Alheshibri M.; Peng Y.; Xie G. Graphite flotation by β-cyclodextrin/kerosene Pickering emulsion as a novel collector. Minerals Engineering 2022, 178, 107412 10.1016/j.mineng.2022.107412.

[ref9] Wang X.; Bu X.; Ni C.; Zhou S.; Yang X.; Zhang J.; Alheshibri M.; Peng Y.; Xie G. Effect of scrubbing medium’s particle size on scrubbing flotation performance and mineralogical characteristics of microcrystalline graphite. Minerals Engineering 2021, 163, 106766 10.1016/j.mineng.2020.106766.

[ref10] Zhou S.; Wang X.; Bu X.; Shao H.; Hu Y.; Alheshibri M.; Li B.; Ni C.; Peng Y.; Xie G. Effects of emulsified kerosene nanodroplets on the entrainment of gangue materials and selectivity index in aphanitic graphite flotation. Minerals Engineering 2020, 158, 106592 10.1016/j.mineng.2020.106592.

[ref11] Chelgani S. C.; Rudolph M.; Kratzsch R.; Sandmann D.; Gutzmer J. A review of graphite beneficiation techniques. Miner. Process. Extr. Metall. Rev. 2015, 37, 58–68. 10.1080/08827508.2015.1115992.

[ref12] Jara A. D.; Betemariam A.; Woldetinsae G.; Kim J. Y. Purification, application and current market trend of natural graphite: A review. Int. J. Min. Sci. Technol. 2019, 671 10.1016/j.ijmst.2019.04.003.

[ref13] Lu X. J.; Forssberg E. Flotation selectivity and upgrading of Woxna fine graphite concentrate. Minerals Engineering 2001, 14, 1541–1543. 10.1016/S0892-6875(01)00168-6.

[ref14] Ni C.; Zhang Q.; Jin M.; Xie G.; Peng Y.; Yu H.; Bu X. Effect of high-speed shear flocculation on the flotation kinetics of ultrafine microcrystalline graphite. Powder Technol. 2022, 396, 345–353. 10.1016/j.powtec.2021.10.041.

[ref15] Li H.; Feng Q.; Yang S.; Ou L.; Lu Y. The entrainment behaviour of sericite in microcrystalline graphite flotation. Int. J. Miner. Process. 2014, 127, 1–9. 10.1016/j.minpro.2013.12.006.

[ref16] Li H.; Ou L.; Feng Q.; Chang Z. Recovery mechanisms of sericite in microcrystalline graphite flotation. Physicochem. Probl. Miner. Process. 2015, 51, 387–400. 10.5277/ppmp150202.

[ref17] Chen Y.; Li S.; Lin S.; Chen M.; Tang C.; Liu X. Promising energy-storage applications by flotation of graphite ores: A review. Chemical Engineering Journal 2023, 454, 139994 10.1016/j.cej.2022.139994.

[ref18] Chen Y.; Truong V. N.; Bu X.; Xie G. A review of effects and applications of ultrasound in mineral flotation. Ultrasonics Sonochemistry 2020, 60, 104739 10.1016/j.ultsonch.2019.104739. 31557697

[ref19] Ozkan S. G. A review of simultaneous ultrasound-assisted coal flotation. J. Min. Environ. 2018, 9, 679–689. 10.22044/jme.2018.6784.1502.

[ref20] Mao Y.; Xia W.; Peng Y.; Xie G. Ultrasonic-assisted flotation of fine coal: A review. Fuel Process. Technol. 2019, 195, 106150 10.1016/j.fuproc.2019.106150.

[ref30] Bu X.; Danstan J. K.; Hassanzadeh A.; Vakylabad A. B.; Chelgani S. C. Metal extraction from ores and waste materials by ultrasound-assisted leaching -an overview. Miner. Process. Extr. Metall. Rev. 2022, 45, 28–45. 10.1080/08827508.2022.2117173.

[ref122] Muthoosamy K.; Manickam S. State of the art and recent advances in the ultrasound-assisted synthesis, exfoliation and functionalization of graphene derivatives. Ultrason. Sonochem. 2017, 39, 478–493. 10.1016/j.ultsonch.2017.05.019. 28732972

[ref21] Letmathe C.; Benker B.; Günther L. Intensification of froth flotation through the use of ultrasound. Aufbereit.-Tech./Miner. Process. 2002, 43, 32–40.

[ref22] Kang W.; Li H. Enhancement of flaky graphite cleaning by ultrasonic treatment. R. Soc. Open Sci. 2019, 6, 191160 10.1098/rsos.191160. 31903206 PMC6936284

[ref23] Li C.; Li X.; Xu M.; Zhang H. Effect of ultrasonication on the flotation of fine graphite particles: Nanobubbles or not?. Ultrason. Sonochem. 2020, 69, 105243 10.1016/j.ultsonch.2020.105243. 32623346

[ref24] Barma S. D.; Baskey P. K.; Rao D. S.; Sahu S. N. Ultrasonic-assisted flotation for enhancing the recovery of flaky graphite from low-grade graphite ore. Ultrasonics Sonochemistry 2019, 56, 386–396. 10.1016/j.ultsonch.2019.04.033. 31101277

[ref25] Chen Y.; Li P.; Bu X.; Wang L.; Liang X.; Chehreh Chelgani S. In-depth purification of spent pot-lining by oxidation-expansion acid leaching – A comparative study. Sep. Purif. Technol. 2022, 303, 122313 10.1016/j.seppur.2022.122313.

[ref26] Wang X.; Shaoqi Z.; Bu X.; Ni C.; Xie G.; Peng Y. Investigation on interaction behavior between coarse and fine particles in the coal flotation using focused beam reflectance measurement (FBRM) and particle video microscope (PVM). Sep. Sci. Technol. 2021, 56, 1418–1430. 10.1080/01496395.2020.1777428.

[ref27] Zhou S.; Wang X.; Bu X.; Wang M.; An B.; Shao H.; Ni C.; Peng Y.; Xie G. A novel flotation technique combining carrier flotation and cavitation bubbles to enhance separation efficiency of ultra-fine particles. Ultrasonics Sonochemistry 2020, 64, 105005 10.1016/j.ultsonch.2020.105005. 32062426

[ref28] Zhou S.; Nazari S.; Hassanzadeh A.; Bu X.; Ni C.; Peng Y.; Xie G.; He Y. The effect of preparation time and aeration rate on the properties of bulk micro-nanobubble water using hydrodynamic cavitation. Ultrasonics Sonochemistry 2022, 84, 105965 10.1016/j.ultsonch.2022.105965. 35240410 PMC8889407

[ref29] Gao J.; Bu X.; Zhou S.; Wang X.; Bilal M.; Hassan F. U.; Hassanzadeh A.; Xie G.; Chelgani S. C. Pickering emulsion prepared by nano-silica particles – A comparative study for exploring the effect of various mechanical methods. Ultrasonics Sonochemistry 2022, 83, 105928 10.1016/j.ultsonch.2022.105928. 35086021 PMC8790493

[ref31] Bu X.; Tong Z.; Bilal M.; Ren X.; Ni M.; Ni C.; Xie G. Effect of ultrasound power on HCl leaching kinetics of impurity removal of aphanitic graphite. Ultrasonics Sonochemistry 2023, 95, 106415 10.1016/j.ultsonch.2023.106415. 37098313 PMC10149312

[ref32] Chen Y.; Chelgani S. C.; Bu X.; Xie G. Effect of the ultrasonic standing wave frequency on the attractive mineralization for fine coal particle flotation. Ultrasonics Sonochemistry 2021, 77, 105682 10.1016/j.ultsonch.2021.105682. 34330084 PMC8329543

[ref33] Ručigaj A.; Connell J. G.; Dular M.; Genorio B. Influence of the ultrasound cavitation intensity on reduced graphene oxide functionalization. Ultrasonics Sonochemistry 2022, 90, 106212 10.1016/j.ultsonch.2022.106212. 36327924 PMC9626748

[ref34] Zhang M.; Xu Z.; Wang L. Ultrasonic treatment improves the performance of starch as depressant for hematite flotation. Ultrasonics Sonochemistry 2022, 82, 105877 10.1016/j.ultsonch.2021.105877. 34920351 PMC8799593

[ref35] Qiang M.; Xiaomin H.; Ke L.; Rui D.; Zhang H.; Bo X.; Kewen Z. Ultrasound-enhanced preparation and photocatalytic properties of graphene-ZnO nanorod composite. Sep. Purif. Technol. 2021, 259, 118131 10.1016/j.seppur.2020.118131.

[ref36] Mao Y.; Chen Y.; Bu X.; Xie G. Effects of 20 kHz ultrasound on coal flotation: The roles of cavitation and acoustic radiation force. Fuel 2019, 256, 115938 10.1016/j.fuel.2019.115938.

[ref37] Chu H.; Chen L.; Lu D.; Wang Y.; Zheng X. Ultrasonic pretreatment of spodumene with different size fractions and its influence on flotation. Ultrasonics Sonochemistry 2022, 82, 105889 10.1016/j.ultsonch.2021.105889. 34979458 PMC8799599

[ref38] Kruszelnicki M.; Hassanzadeh A.; Legawiec K. J.; Polowczyk I.; Kowalczuk P. B. Effect of ultrasound pre-treatment on carbonaceous copper-bearing shale flotation. Ultrasonics Sonochemistry 2022, 84, 105962 10.1016/j.ultsonch.2022.105962. 35259571 PMC8904613

[ref39] Mao Y.; Bu X.; Peng Y.; Tian F.; Xie G. Effects of simultaneous ultrasonic treatment on the separation selectivity and flotation kinetics of high-ash lignite. Fuel 2020, 259, 116270 10.1016/j.fuel.2019.116270.

[ref40] Wang W.; Liu D.; Tu Y.; Jin L.; Wang H. Enrichment of residual carbon in entrained-flow gasification coal fine slag by ultrasonic flotation. Fuel 2020, 278, 118195 10.1016/j.fuel.2020.118195.

[ref41] Swamy K. M.; Narayana K. L., Ultrasonically assisted leaching, In Mason T. J.; Tiehm A. (Eds.) Advances in Sonochemistry: Ultrasound in Environmental Protection; JAI Press: Tokyo, Japan, 2001.

[ref42] Bu X.; Xie G.; Chen Y.; Ni C. The order of kinetic models in coal fines flotation. International Journal of Coal Preparation & Utilization 2017, 37, 113–123. 10.1080/19392699.2016.1140150.

[ref43] Sokolović J. M.; Miskovic S. The effect of particle size on coal flotation kinetics: A review. Physicochem. Probl. Miner. Process. 2018, 54, 1172–1190. 10.5277/ppmp18155.

[ref44] Filippov L. O.; Filippova I. V.; Barres O.; Lyubimova T. P.; Fattalov O. O. Intensification of the flotation separation of potash ore using ultrasound treatment. Miner. Eng. 2021, 171, 107092 10.1016/j.mineng.2021.107092.

[ref45] Cao D.; Xu X.; Jiang S. Ultrasound-electrochemistry enhanced flotation and desulphurization for fine coal. Sep. Purif. Technol. 2021, 258, 117968 10.1016/j.seppur.2020.117968.

[ref46] Ozkan S. G.; Kuyumcu H. Z. Investigation of mechanism of ultrasound on coal flotation. Int. J. Miner. Process. 2006, 81, 201–203. 10.1016/j.minpro.2006.07.011.

[ref47] Jin L.; Wang W.; Tu Y.; Zhang K.; Lv Z. Effect of ultrasonic standing waves on flotation bubbles. Ultrasonics Sonochemistry 2021, 73, 105459 10.1016/j.ultsonch.2020.105459. 33621851 PMC7905344

[ref48] Chen Y.; Ni C.; Xie G.; Liu Q. Toward efficient interactions of bubbles and coal particles induced by stable cavitation bubbles under 600 kHz ultrasonic standing waves. Ultrasonics Sonochemistry 2020, 64, 105003 10.1016/j.ultsonch.2020.105003. 32062535

[ref49] Chen Y.; Zheng H.; Truong V. N. T.; Xie G.; Liu Q. Selective aggregation by ultrasonic standing waves through gas nuclei on the particle surface. Ultrasonics Sonochemistry 2020, 63, 104924 10.1016/j.ultsonch.2019.104924. 31945565

[ref50] Zhu L.; Lyu W.; Yang P.; Wang Z. Effect of ultrasound on the flocculation-sedimentation and thickening of unclassified tailings. Ultrasonics Sonochemistry 2020, 66, 104984 10.1016/j.ultsonch.2020.104984. 32247237

